# Strongyloides Hyperinfection and Miliary Tuberculosis Presenting with Syndrome of Inappropriate Antidiuretic Hormone Secretion in a Malnourished Patient

**DOI:** 10.7759/cureus.2349

**Published:** 2018-03-20

**Authors:** Arjun Saradna, Amith Shenoy, Paurush Ambesh, Stephan Kamholz

**Affiliations:** 1 Internal Medicine, Maimonides Medical Center; 2 Pulmonary Critical Care, Winthrop University Hospital; 3 Chair, Department of Medicine, Maimonides Medical Center

**Keywords:** strongyloides, tuberculosis, hyperinfection, siadh, stercoralis, miliary

## Abstract

Strongyloides stercoralis (S. stercoralis) is an intestinal nematode endemic to tropical regions. An accelerated infection, known as a hyperinfection, occurs in immunocompromised patients, most commonly those treated chronically with glucocorticoids or those who have human T cell leukemia virus-1 (HTLV-1) infection. We describe a 67-year-old Hispanic female who presented with complaints of decreased oral intake and fatigue since three months. Hyponatremia on initial presentation was attributed to syndrome of inappropriate antidiuretic hormone (SIADH) secretion and managed with fluid restriction. Computed tomography (CT) of the chest revealed multiple pulmonary nodules suggestive of miliary tuberculosis, however, sputum acid-fast bacilli (AFB) smears were negative. Fiberoptic bronchoscopy with bronchoalveolar lavage (BAL) was performed and specimens sent for AFB testing. A concurrent endoscopy with biopsy was done to evaluate dysphagia. Both respiratory and gastrointestinal (GI) specimens were positive for Strongyloides stercoralis. Treatment with ivermectin and prophylactic antibiotics was started. The patient developed septic shock and had multiple episodes of gastrointestinal bleeding. Despite aggressive management, she expired. Subsequently, cultures for Mycobacterium tuberculosis (MTB) were positive and the autopsy demonstrated evidence of MTB infection in the lungs, liver, and lymph nodes. This case illustrates the importance of considering co-infection with Strongyloides stercoralis in patients with MTB, both associated with depressed cellular immunity.

## Introduction

Strongyloides stercoralis (S. stercoralis) is a pathogenic intestinal nematode. Manifestations range from the absence of symptoms to fatal disseminated disease. Strongyloides stercoralis can produce autoinfection, characterized by the ability of non-infective rhabditiform larvae to transform into infective filariform larvae in the human intestine, which then reinfect the same host. Autoinfection allows S. stercoralis to persist in the host for decades after the initial infection [1]. After infection in immunocompetent hosts, about 50% of them remain asymptomatic while the others have mild gastrointestinal or pulmonary symptoms. In immunocompromised hosts, acutely accelerated multiplication of the parasite occurs within the gastrointestinal tract, referred to as hyperinfection [2].

Hyperinfection can lead to the involvement and damage of various organs, with a disseminated infection, which may be fatal despite optimal therapy. Tuberculosis is well known to be associated with an immunosuppressed state. The reactivation of tuberculosis occurs during episodes of reduced immune surveillance, such as stress, malnutrition, infections, and medications. Concurrent tuberculosis and Strongyloides hyperinfection are rarely reported, with only two prior case studies in the literature [3-4]. Our 67-year-old female patient was found to have tuberculosis and developed a Strongyloides stercoralis hyperinfection.

## Case presentation

A 67-year-old Hispanic woman presented to the hospital, complaining of progressively worsening fatigue since three months and decreased oral intake for 10 days. Oral intake was reported to have decreased due to abdominal pain and burning sensation in her throat associated with swallowing. She had been diagnosed with breast cancer in 2002 for which she had surgery but refused chemotherapy. Other medical history included a diagnosis of vitamin B12 deficiency, peptic ulcer disease, and left hip arthroplasty at the age of 64. She was born in Mexico but emigrated to the United States at the age of 37.

The patient had a prior admission for the same complaint at a different facility two weeks ago during which an endoscopy was performed for the evaluation of anemia, which had revealed pre-pyloric peptic ulcer disease.

Upon initial assessment, the patient appeared frail and older than the stated age. There was mild temporal wasting, mucous membranes were dry, and a lung examination revealed bilateral lower lung field crackles. The abdomen was soft and non-distended with mild diffuse tenderness, but no guarding, rebound, or palpable organomegaly.

The vital signs were: blood pressure 119/66 mmHg; pulse 121 per/min; oxygen saturation 96% (room air); and respiratory rate 22/min; white blood cell count 7.9K/μL, with 72% neutrophils, 15% band neutrophils, and 3% eosinophils; sodium 121 mmol/L; calcium 7.2 mg/dL; and serum albumin 1.8 g/dL. Prothrombin time and partial thromboplastin time were slightly prolonged and b-type natriuretic peptide (BNP) was 1905 pg/ml. Liver function tests were within the normal range except for a mild elevation of alkaline phosphatase. The chest roentgenogram revealed a seven millimeter (mm) nodular density projecting over the right lower lung field. The transthoracic echocardiogram (ECHO) demonstrated a left ventricular ejection fraction (EF) of 60%-65% with no valvular vegetations.

Further laboratory assessment upon admission included urine osmolarity of >100 millimoles per liter (mmol/L) and urine sodium of 96 mmol/L. Plasma osmolarity was normal, indicative of euvolemic hyponatremia. Syndrome of inappropriate antidiuretic hormone (SIADH) secretion was diagnosed in the context of the history of breast cancer and the newly identified lung nodule. Thyroid stimulating hormone and cortisol levels were within the normal range. One liter daily fluid restriction was initiated, followed by improvement in serum sodium. Subsequently, serum sodium levels remained in the 120 meq/L range. Intravenous hydration with half normal saline and half sodium bicarbonate was initiated, resulting in an improvement in hyponatremia.

**Figure 1:**
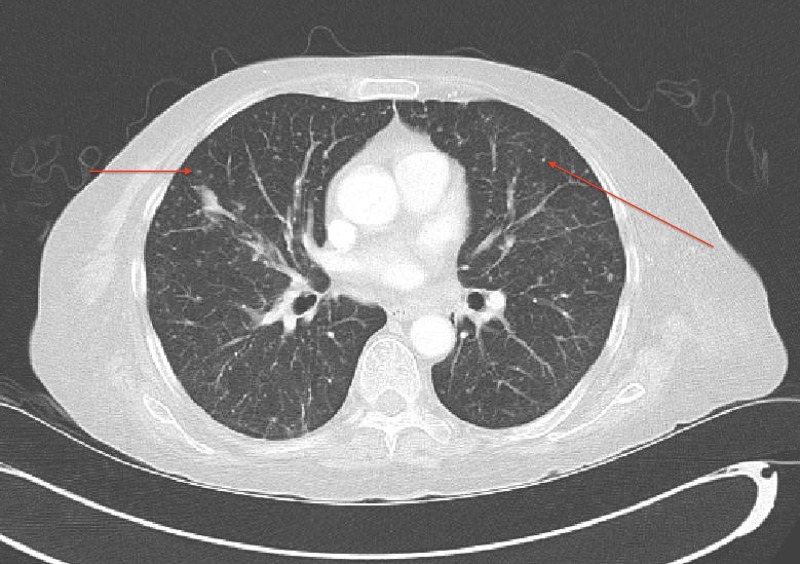
Computed tomography scan of the chest showing multiple small, bilateral pulmonary nodules (red arrows) consistent with miliary tuberculosis

Endoscopic results from prior hospitalization were reviewed, which demonstrated pre-pyloric ulcers, white plaques over the esophagus, stomach, and duodenum. The biopsy result from the plaques was reported on day three of the current admission to be positive for Strongyloides stercoralis. Computed tomography (CT) of the chest, abdomen, and pelvis demonstrated >100 small bilateral pulmonary nodules (Figure 1); mediastinal, axillary, and retroperitoneal necrotic lymphadenopathy, thickening of the distal transverse colon, and a possible lytic lesion of the left pubic symphysis. The patient was placed in airborne isolation for possible miliary tuberculosis. Serial sputum acid-fast bacilli (AFB) stains were negative and cultures were established. The Quantiferon test was not positive. The stool specimen demonstrated Strongyloides species. Ivermectin 9 milligrams (mg) every 24 hours was initiated along with fluconazole to treat Strongyloides stercoralis and Candida esophagitis. Piperacillin with tazobactam was administered to prevent gram-negative bacteremia, which is often associated with parasite migration. Interventional radiology (IR) guided biopsy of the largest lung nodule was performed on day five of admission, which showed non-caseating granuloma, without demonstrable acid-fast bacilli. Soon thereafter, the patient developed hypotension in the setting of septic shock and was transferred to the medical intensive care unit (MICU) for intensive management, including vasopressors and broad-spectrum antibiotics. Mechanical ventilation was initiated due to the development of acute respiratory failure. The patient deteriorated further with the development of disseminated intravascular coagulation and had multiple episodes of gastrointestinal bleeding. Endoscopy revealed multiple gastric ulcers with clots in a duodenal diverticulum. Embolization of the bleeding vessel was performed. Bronchoscopy lavage was performed and demonstrated Strongyloides larvae but AFB stains were negative. Her course became complicated with multiple organ involvement, and she expired on hospital day 15. Twelve days later, respiratory cultures yielded Mycobacterium tuberculosis complex. The autopsy revealed miliary Mycobacterium tuberculosis involving both lungs, para-tracheal and periaortic lymph nodes, the liver, and the pericolic soft tissue.

## Discussion

Strongyloides stercoralis is an intestinal parasite endemic to Latin America, Africa, and parts of Asia. It is less commonly encountered in the United States with the highest incidence in the southeastern states and in the immigrant population from endemic areas. Chronic infection with S. stercoralis can persist indefinitely and is perpetuated by the mechanism of autoinfection. Our patient immigrated to the US from Mexico, which is an endemic area for S. stercoralis, at the age of 37. It is likely that she had a chronic infection because she did not travel to endemic areas after arriving in the US.

Humans acquire infection through the transcutaneous route. The infectious filariform larvae of S. stercoralis penetrate the skin and then travel via the bloodstream to the lungs, ascend the tracheobronchial tree, and are then swallowed, entering the gastrointestinal tract. Filariform larvae undergo molting in the small intestine, becoming rhabditiform larvae, which are excreted in feces and then molt into filariform larvae in soil [5]. Sometimes, the rhabditiform larvae in the intestine become filariform larvae, penetrate the intestinal mucosa, and reinfect the same host; this is known as autoinfection.

Autoinfection is minimized by an intact immune system. A low level of autoinfection allows the organism to persist in the same host for decades. Diminished host immunity leads to the increased rapidity of autoinfection cycles, resulting in a markedly increased load of infectious filariform larvae production. These filariform larvae can disseminate to various organs, causing end-organ damage. This phenomenon is called hyperinfection. Conditions that predispose to hyperinfection include corticosteroid therapy [6], human T-cell leukemia-1 virus infection, malnutrition [7], hematological malignancy, kidney and bone marrow transplant, immunosuppressive medications (vincristine and cyclosporin), and human immunodeficiency virus (HIV) infection. These factors were ruled out in our patient during hospitalization.

There are only two prior reports of tuberculosis as a potential trigger for Strongyloides hyperinfection. Our patient was found to have disseminated tuberculosis infection, which may have predisposed to a Strongyloides hyperinfection or possibly the Strongyloides hyperinfection could have triggered a reactivation of latent tuberculosis. Both Strongyloides and tuberculosis are associated with impaired cellular immunity. During the initial T cell response to a Mycobacterium tuberculosis infection, the expansion of Foxp3 regulatory T cells occurs, which delays the onset of adaptive immunity. Regulatory T cell expansion is associated with the impairment of the immune response to Strongyloides infection in mouse models [8-9]. In patients with decreased cell-mediated immunity, case fatality rates from hyperinfection are reportedly 50%-86%.

Peripheral blood eosinophilia is an important marker of helminthic infection but may not occur in Strongyloidiasis. Previous reports suggest that patients who have an absence of eosinophilia in the setting of a Strongyloides infection tend to have a poorer prognosis. The reason for this observation is unclear, but it may be related to corticosteroid-induced neutrophilia, and corticosteroid therapy is an important trigger for a Strongyloides hyperinfection. Corticosteroids can promote apoptosis of eosinophils. Our patient did not manifest peripheral eosinophilia and had not received chronic steroid therapy.

Our malnourished patient had lymphopenia (4% on differential leukocyte count). Malnutrition may be associated with reduced lymphocyte count, and malnutrition is, in itself, an important risk factor for a Strongyloides hyperinfection. Malnutrition in this patient was manifested by temporal wasting and hypoalbuminemia (1.8 g/dL). Malnutrition may have been the trigger leading to decreased lymphocyte count, which increased the risk for tuberculosis reactivation and Strongyloides hyperinfection.

SIADH has been associated with Strongyloides hyperinfection in the past. The mechanism is unknown, although it has been attributed to pulmonary or central nervous system (CNS) involvement. One report attributed SIADH to anorexia [10]. Our patient had SIADH at the time of admission. Hyponatremia was resistant to fluid restriction. Alternatively, miliary pulmonary tuberculosis could have triggered SIADH.

In addition to extensive pulmonary involvement, this patient had repeated episodes of gastrointestinal bleeding. Severe gastrointestinal bleeding with Strongyloides hyperinfection occurs due to a massive larval invasion of gastrointestinal mucosa, which increases the friability and sloughing of the mucosa. This leads to an increased risk of gram-negative bacterial translocation from the gut into the bloodstream. Other mechanisms that are thought to promote bacterial infection in the setting of the hyperinfection syndrome include small bowel bacterial overgrowth and bacterial adherence onto the larval surface. Patients with Strongyloides hyperinfection should receive empiric antimicrobial therapy to reduce the likelihood of gram-negative bacteremia.

## Conclusions

This report describes a patient with a concurrent Strongyloides stercoralis hyperinfection and a miliary Mycobacterium tuberculosis infection, highlighting the similarities among triggers, cellular responses, and the co-existence of these two infections. The possibility of a second infection should be suspected in patients with impaired cellular immunity.
